# Comparative Analysis of Alpha and Beta HPV E6 Oncoproteins: Insights into Functional Distinctions and Divergent Mechanisms of Pathogenesis

**DOI:** 10.3390/v15112253

**Published:** 2023-11-14

**Authors:** Josipa Skelin, Vjekoslav Tomaić

**Affiliations:** Division of Molecular Medicine, Ruđer Bošković Institute, Bijenička 54, 10000 Zagreb, Croatia; jskelin@irb.hr

**Keywords:** alpha-HPV, beta-HPV, E6 oncoprotein, cervical cancer, HNSCC, cSCC

## Abstract

Human papillomaviruses (HPVs) represent a diverse group of DNA viruses that infect epithelial cells of mucosal and cutaneous tissues, leading to a wide spectrum of clinical outcomes. Among various HPVs, alpha (α) and beta (β) types have garnered significant attention due to their associations with human health. α-HPVs are primarily linked to infections of the mucosa, with high-risk subtypes, such as HPV16 and HPV18, being the major etiological agents of cervical and oropharyngeal cancers. In contrast, β-HPVs are predominantly associated with cutaneous infections and are commonly found on healthy skin. However, certain β-types, notably HPV5 and HPV8, have been implicated in the development of non-melanoma skin cancers in immunocompromised individuals, highlighting their potential role in pathogenicity. In this review, we comprehensively analyze the similarities and differences between α- and β-HPV E6 oncoproteins, one of the major drivers of viral replication and cellular transformation, and how these impact viral fitness and the capacity to induce malignancy. In particular, we compare the mechanisms these oncoproteins use to modulate common cellular processes—apoptosis, DNA damage repair, cell differentiation, and the immune response—further shedding light on their shared and distinct features, which enable them to replicate at divergent locations of the human body and cause different types of cancer.

## 1. Introduction

Human papillomaviruses (HPVs) are a family of small DNA viruses that infect epithelial cells at various anatomical sites of the human body. It is a large and diverse group of viruses, divided into five genera (alpha (α), beta (β), gamma (γ), mu (μ), and nu (ν)) based on the open reading frame sequence coding for the L1 capsid protein. Thus far, 451 different HPVs have been discovered [1,2,3]. Among the five genera, α and β have received the most comprehensive attention, largely because of their capacity to pose health risks to humans. 

Based on the ability to cause cancer, α-HPVs can be classified as low-risk (LR) and high-risk (HR) types. Infections with LR types, like HPV-6 and -11, result in benign anogenital warts, while HR types, such as HPV-16 and -18, contribute to HPV-induced carcinogenesis in a subset of infected individuals [4,5,6]. HPV-16 and -18 alone account for roughly 80% of cervical cancer (CC) cases, with other HR types being associated with the remaining 20% [4,5]. HR mucosal α-HPVs are also implicated in approximately 70% of other anogenital cancers and up to 50% of head-and-neck squamous cell carcinomas (HNSCC), where HPV-16 is the most prevalent cancer-causing type [7]. The malignant cellular transformation driven by HR-HPV oncoproteins is predominantly caused by the process of integration of viral genomes into the host genome, leading to the deregulation of oncoprotein expression and the abrogation of the productive viral life cycle [8]. 

Unlike α-HPVs, HPVs of the β genus are not carcinogenic in themselves but can contribute to the development of skin cancer in specific cases, such as in patients with the genetic disorder Epidermodysplasia verruciformis (EV) or those who are immunocompromised [9,10]. β-HPV types, such as HPV-5 and HPV-8, have been found in EV patient skin lesions and squamous cell carcinoma (SCC) samples; however, subsequent analyses have shown that β-HPVs do not cause cancer but facilitate its occurrence by inhibiting apoptosis in response to UV damage [11,12]. Additionally, and importantly, β-HPV oncoproteins do not integrate into the host genome. 

Most of the current knowledge about the virus was acquired from either tissue samples or various cellular and transgenic animal models of HPV-associated disease. Clinical samples are mostly used in epidemiological studies and biomarker discovery and validation but cannot be propagated indefinitely *in vitro*. For this reason, as well as for consistency and reproducibility, HPV-expressing keratinocytes are frequently used in monolayer and 3D organotypic raft cultures [13]. This approach is particularly useful for investigating the process of early infection, the viral life cycle of HR-HPVs, and malignant transformation, in addition to allowing the expression of individual viral proteins and evaluating their interactome [14,15]. For LR and cutaneous HPVs, which seem to persist less efficiently in organotypic raft culture systems, animal mouse models are used as an efficient way to circumvent this [13]. A good example is MmuPV1, the recently discovered murine naturally occurring papillomavirus. Since infected mice with this virus can carry the complete viral productive life cycle in both cutaneous and mucosal tissues, eventually resulting in malignant development, it is considered an excellent model for studying various viral aspects [16]. Furthermore, another commonly used transgenic mouse model is based on genetically engineered mice expressing individual or combined HPV genes under keratin promoters, such as HPV8 early genes or HPV16 oncoproteins, being expressed under a human keratin 14 promoter [17,18,19]. In this way, various HPV protein functions can be dissected. In addition, recent reports indicate that a direct, anatomically local transfection with HPV-gene-expressing plasmids can be used to obtain a spontaneous model of cervical carcinogenesis [20,21]. Such mouse models can be used to examine the progression of infection *in vivo*, to evaluate the role of cellular factors in HPV-associated disease, and to determine the individual impacts of each HPV oncoprotein.

**Figure 1:**
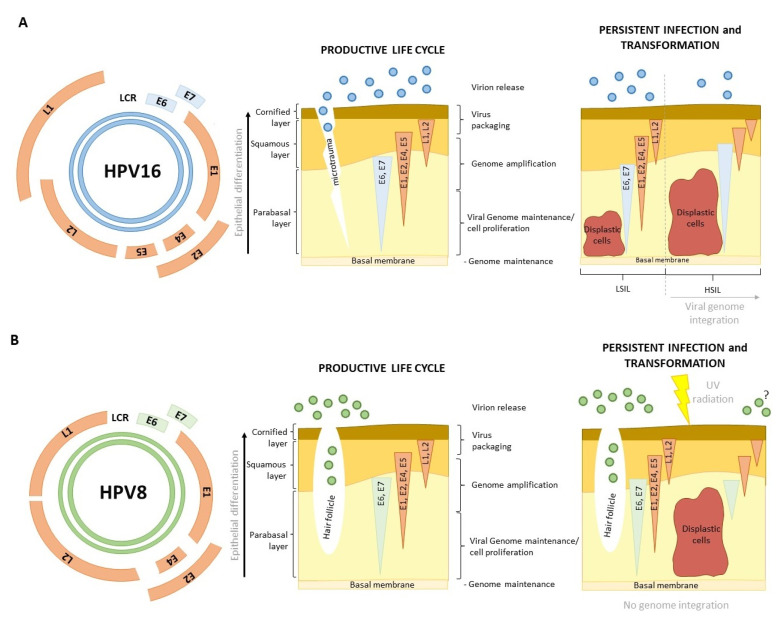
Differences in genome organization, viral life cycle, and malignant progression between α- and β-HPVs. (**A**) HR-HPVs infect mucosal epithelia via micro-wounds, and they manipulate differentiating cells to efficiently propagate new virions, with viral gene expression (light blue—oncoproteins, orange—other early proteins and capsid proteins) coinciding with the stages of keratinocyte maturation (middle panel). As basal cells become dysplastic, the viral gene expression becomes more disordered, while E6/E7 acquire the primacy in expression over other proteins. This process leads to a reduced productive infection, resulting in the release of fewer virions (right panel). Premalignant stages are also indicated (LSIL—low-grade squamous interepithelial lesion; HSIL—high-grade squamous interepithelial lesion). (**B**) The natural reservoir for HPV8 and many other β-types is considered to be cutaneous epithelia, including hair follicles. Their life cycle is also dependent on keratinocyte differentiation, with orchestrated expression of viral genes delaying keratinocyte maturation and enabling the production of new virions (middle panel). β-HPV genomes do not integrate into the host genome and are considered to act as cofactors in tumor initiation but are not necessary for tumor maintenance. The viral load is higher in premalignant states than in cSCC, and it is currently unknown whether β-HPVs can complete their life cycle in transformed cells (right panel). Expanded and adapted from [23].

E6 and E7 oncoproteins are essential for supporting persistent infection and viral propagation of both α- and β-HPVs, with α-HPVs coding for an auxiliary pseudo oncoprotein E5 that is also involved in this process [22]. Specifically, E6 and E7, two major HPV oncoproteins, are required for cellular reprogramming needed for optimizing the environment, which is necessary for undisturbed viral replication, but importantly, they also play crucial roles in HPV-driven malignancies (Figure 1) [23]. E6 oncoproteins of both α- and β-HPVs target some of the same cellular pathways, but usually in unique ways. A good example of this would be various ways of targeting p53 by α and β-E6s, ultimately resulting in disruption of p53 normal activities [24]. Furthermore, E6 oncoproteins from both genera aim to disrupt the normal processes of apoptosis and cell differentiation, as well as the DNA repair system, in order to ensure the continuous proliferation of cells, the replication of the viral genome, and cell survival [25]. However, these E6 activities are not solely sufficient to immediately drive cellular transformation; rather, all of the new traits’ cells acquire as a consequence of E6 oncoprotein long-term expression, together with other HPV-dependent modulations, are later likely to contribute to the malignant phenotype [26,27]. In α-HPV-induced cancers, E6 was shown to be indispensable for the maintenance of this phenotype. This was supported by previous studies, which have shown that interfering with E6 at either RNA or protein levels can be an efficient way of pushing HPV-positive (HPV+) cells into senescence or apoptosis [28,29]. Therefore, understanding the disparities in E6 functions is crucial for developing targeted prevention and direct treatment strategies, which are currently still not available. 

## 2. Differences in Genome Organization

The HPV genome is approximately 8 kb long and organized into the early (E) region, the late (L) region, and the upstream regulatory region (URR), where the origin of DNA replication is located. The E region typically contains six open reading frames (ORFs): E1, E2, E4, E5, E6, and E7, while the L region contains two: L1 and L2 (Figure 1A) [30,31]. The early genes, E1 and E2, and the late genes, L1 and L2, are well conserved between the genera. The E1 gene encodes a virus-specific DNA helicase, which is crucial for the replication and amplification of the viral genome. Additionally, it can trigger breaks in the host DNA and activate the ATM (Ataxia–Telangiectasia Mutated) damage response pathway, which is important for HPV DNA replication [32]. The E2 protein serves as a viral transcription factor and binds to sites in both the host and viral DNA. Additionally, E2 enhances the binding of E1 to the origin of replication [33]. It also regulates E6 and E7 expressions at the transcriptional level, keeping these viral transcripts at low copy numbers until the infected keratinocytes undergo differentiation [34]. L1 and L2 are major and minor capsid proteins whose conservation is important for proper virion assembly and proper encapsidation of the viral genomes. 

In comparison to L1/L2 and E1/E2, there are many more variations present among the other early genes. The E4 protein, involved in viral maturation and escape from the cell surface, can be found in all HPV genera, but interestingly, it is poorly conserved [35]. This variability is thought to reflect the adaptation of individual species to the tissue they preferentially infect. The heterogeneity of viral genes also extends to E6 and E7, even though there is considerable conservation of important functional domains, especially concerning the E7 oncoprotein [36,37,38]. It is important to note that the variations in the viral genome sequence exist below the level of HPV type. These genomic nucleotide differences of 1–10% are called variants, and they have a specific geographic and ethnic distribution [31,39,40,41]. Several studies have addressed the differences in functional abilities between different HR E6 variants, and some of them were indeed found to be more efficient in target degradation or interaction [42,43]. What is also interesting is that some variants were found to be present in higher frequencies in particular sites of infection. For example, 16 E6 variant R10G is rarely detected in CC but was found to be relatively frequent (19%) in tonsillar SCC [44]. Detailed structural and functional analyses still have not completely elucidated this phenomenon, but it seems that even minute changes in the E6 gene sequence could be critical [45]. 

One important difference between α- and β-HPV genomes is that β-types lack the ORF for the E5 oncoprotein (Figure 1). E5 is a small (between 40 and 93 amino acids (aa) long), triple-pass, hydrophobic transmembrane protein, which can oligomerize to create transmembrane channels [46,47]. Interestingly, β-HPVs, γ-, and μ-genera also lack E5. Even within the α-genus, the E5 gene seems to be highly variable and can thus be phylogenetically classified into four separate families, termed α to δ [48]. All HR-HPVs share the E5α protein, which has a variety of roles within infected keratinocytes [48]. It ensures the replication of differentiating keratinocytes by enhancing growth pathways [49]. It can alter signal transcription and transduction by binding hub-proteins [22], modulate the immune response by manipulating the transcription of MHC I (Major Histocompatibility Complex I) molecules [50], and apoptosis by reducing receptor transcription [22,51]. Notably, several studies have found that E5 can induce the transformation of fibroblasts and epithelial cells, probably by enhancing EGFR (Epidermal Growth Factor Receptor) signaling [52,53,54]. Nevertheless, the E5 protein of mucosal α-HPVs is considered to be lost during viral genome integration and cellular transformation, even though E5 gene transcripts have been found in high-grade lesions and CC samples. This could be due to a heterogeneous population of cancer cells in some of which the viral genome integration was not complete, and in this case, E5 could potentially contribute to cell transformation [55,56,57]. This could be of importance in HNSCC, where the HPV genome was found to be integrated in only 50–60% of cases [58]. 

E5 plays a major role in adapting the cellular environment for viral replication in the cases of mucosal HPV infections. It is possible that its role is indispensable for viral replication in this specific type of tissue and that it was lost in cutaneous species because it did not provide an evolutionary advantage. Even though it clearly is not crucial for all HPV genera, its functions definitely contribute to the initial stages of HR-HPV-induced carcinogenesis. 

## 3. Tissue Tropism

In addition to their host and the sequence of the L1 ORF, papillomaviruses can also be classified based on their infection preference for a particular anatomical site. The α-HPV genus contains species that infect both mucosal and cutaneous epithelia. Interestingly, LR α-HPVs are capable of infecting both mucosal and cutaneous tissues, while all HR α-HPVs exclusively infect and replicate in mucosal epithelia. β-types, on the other hand, are considered cutaneous; the infection is usually subclinical, and papillomas appear only in immunocompromised individuals [59,60]. 

What is particularly interesting is that even different HR α- types show varying capabilities for infecting keratinocytes of different anatomical sites. For example, 70% of CC cases are caused by HPV16 and HPV18, while nearly all HPV+ HNSCC are caused by HPV16 [59,61]. Similarly, the LR α-HPV type HPV6 causes warts primarily in genital sites, while a very similar HPV11 preferentially infects oral sites [59,62,63]. The preference for a specific anatomical site depends on multiple factors, including viral genotype and host immunity. Current research suggests that viral entry plays less of a part in tissue tropism than viral gene expression and that regulatory elements within the LCR play a pivotal role in determining the range of tissues that the virus can infect [59,64,65]. One additional factor in tropism could also be the virion surface charge, as differences were reported between cutaneous and mucosal species [66]. Recent advancements in detection methods have facilitated the detection of even greater numbers of papillomaviruses isolated from hair follicles, skin, and genital lesions, and this has put into question the strict division in tissue preference between HR α-HPVs and β-HPVs [67]. Dual tropism has been confirmed for the most common cutaneous β-HPV species, β-1 and β-2, which have been found in the oral and anal mucosa, as well as the nasal cavity [68,69]. Additionally, the three HPV types of the β-3 group, the group most similar to HR α-HPVs, have been found to be able to immortalize or extend the life span of human foreskin keratinocytes [70]. The ability of these different β-types to complete their life cycle in mucosal cells has not yet been proven, and neither has the potential influence of their presence on cell behavior. 

It is uncommon for diseases caused by specific HPV types to manifest at atypical sites, such as HPV1, causing warts in sites other than hands and feet [71]. When such instances do occur, they often present with non-standard characteristics in terms of morphology and pathology. This indicates that while the anatomical site might accommodate viral infection, it might not be adapted or adaptable to viral propagation. That being said, researchers investigating viral tropism should take note and not limit themselves to a single body region or anatomic site.

## 4. The HPV Life Cycle 

The viral life cycle is perfectly coordinated with the process of keratinocyte differentiation. This process begins in the basal layer of the epithelium, where stem cells reside. In normal tissue development, when basal cells divide, one resulting daughter cell remains in the basal layer, while the other begins the process of terminal differentiation and starts to move toward the top layers of the stratified epithelium. As keratinocytes ascend, they enter the spinous layer, where structural proteins such as involucrin, loricrin, and various cytokeratins critical for cellular cohesion and the development of the cornified envelope begin to be produced. Following this, cells enter the granular layer, where intracellular lipids are accumulated, making the skin impermeable to water. Finally, keratinocyte differentiation is finalized in the stratum corneum [72].

HPV infection begins when the virus gains access to the basal cells, often via a micro injury or, in some cases, as with cutaneous HPVs, via hair follicles (Figure 1) [73]. HPVs gain entry into cells via endocytosis. The penetration of the cell, facilitated by L1 and L2, involves interactions with various cellular components such as heparan sulfate and proteoglycans; however, the precise cellular receptor responsible for this process remains unidentified [60]. Once the infection occurs with a limited number of virions, HPVs begin to replicate independently of the cell cycle, resulting in the production of approximately 50–100 viral copies within each cell, where they exist as episomal DNA. This autonomous viral replication is driven by the expression E1 and E2 [32,34]. The relatively slow rate of viral replication in basal cells serves to evade immune system detection and response [50]. This initial process of infection is thought to be the same for both α- and β-HPVs (Figure 1). After the autonomous genome replication during early infection, the viral life cycle becomes completely dependent on keratinocyte differentiation (Figure 1). 

The process of late HPV infection and its synchronization with keratinocyte differentiation is fairly well-defined for α-HPVs but still somewhat unclear for β-HPV types. As basal cells divide, commit to the keratinocyte lineage, and travel vertically through the skin, they carry HPV genomes with them. E6 and E7 oncoproteins start being expressed at this time, initially to ensure that the differentiating cells remain in the cell cycle (E7) and that they do not succumb to apoptosis as a consequence of proliferation (E6). The ability to force proliferation in terminally differentiating cells seems to be a key characteristic of HR-HPVs, even though many other HPV types can drive cell cycle re-entry [9]. E1^∧^E4 also starts to be expressed at this time. E4 contributes to genome amplification and disrupts keratin filaments within the cell to mitigate virion release. For this reason, its expression is increased toward the top layers of epithelia [35]. Finally, E2 is again expressed from the late promoter, which leads to E6 and E7 downregulation, allowing for the terminal differentiation of the cells and the expression of HPV capsid proteins. After this, the virions are assembled and shed with the dead keratinocytes (Figure 1A) [74]. 

HPV infections are usually asymptomatic and cleared by the immune system but can also result in neoplasia. In the case of HR α-HPVs, cellular transformation occurs as a result of oncoproteins’ dysregulation (Figure 1A). This usually happens after the process of integration of viral genomes into the host genome, which results in the loss of all genes except E6 and E7 [75,76]. In the case of β-HPVs, the process is not nearly as clear. β-HPVs are a part of the microbiota of 80–90% of individuals, but their role in skin carcinogenesis is still being debated (Figure 1B). Most researchers agree that β-HPVs can act as cofactors in carcinogenesis, an opinion supported by several meta-studies claiming that β-1 and β-2 seropositivity in healthy individuals indicates a higher risk of developing cSCC, as well as numerous studies about the impact of β-HPVs on skin carcinogenesis in immunocompromised individuals [77,78,79]. The currently suggested mechanism claims that the expression of β-HPV oncoproteins in skin keratinocytes alters the local immune response, prolongs the process of differentiation, interferes with DNA damage repair and apoptosis, and that all of this makes the cell susceptible to UV damage [80,81,82,83]. Unlike α-HPVs, where viral integration drives malignant transformation, β-HPVs are cofactors in the process (Figure 1B). The UV damage induces mutations that accumulate and eventually lead to cellular transformation [79]. Once the cell becomes malignant, β-HPV oncoproteins are no longer needed to maintain the phenotype, unlike in α-HPV-induced cancers (Figure 1) [27].

## 5. E6 Oncoproteins and the Commonly Targeted Pathways

**Figure 2:**
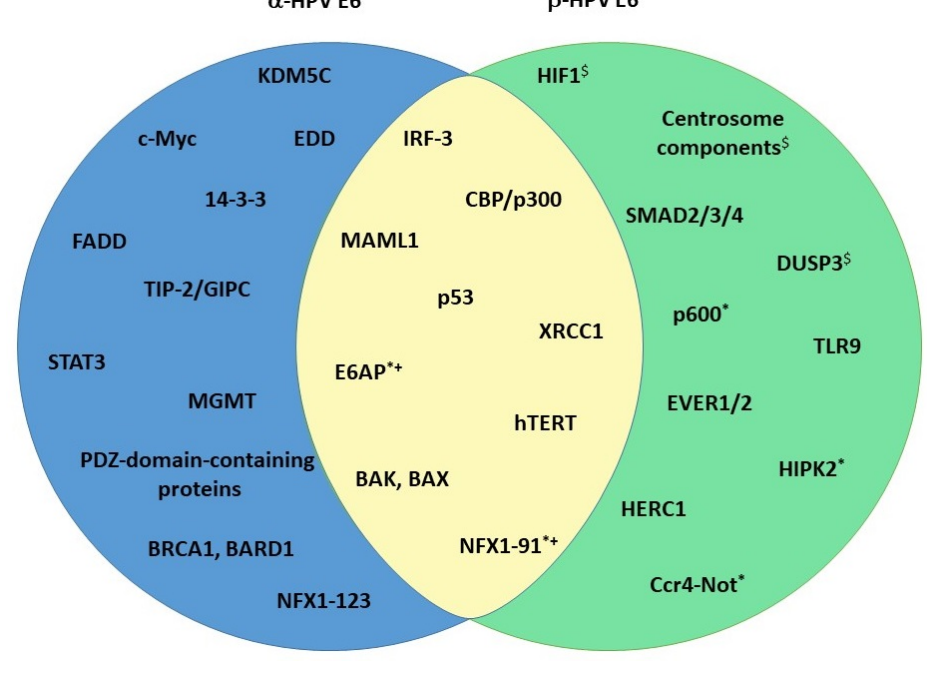
The most important binding partners and target substrates of E6 oncoproteins. α-E6 exclusive interacting partners are indicated in the blue circle, β-E6 specific ones in the green circle, and common ones in the yellow overlap. * denotes that the protein is a partner of the β2 species, ^+^ refers that the protein is a partner of the β3 species, and ^$^ refers that the protein is the partner of β4 species.

As described above, E6 is one of the two crucial HPV oncoproteins. It is about 150 amino acids (aa) long and has a mass of about 18 kDa. Structurally, it contains two zinc-finger domains with two C-x-x-C motifs, each connected with a short linker helix [84]. The two zinc-finger domains form a hydrophobic pocket that binds proteins containing an acidic LXXLL motif [85]. Both of the zinc-binding domains are important for E6 structural integrity, and they are both crucial for E6 function [86]. The two zinc-binding domains are conserved between E6 oncoproteins of different genera. On the C-terminal of HR-α-E6 oncoproteins, there is a PDZ-binding motif (PBM) that enables the virus to interact with PDZ domain-containing proteins (will be discussed later) [87]. Because the PBM can be found only on E6 oncoproteins of cancer-causing HPVs, it is considered to be one of the pivotal factors responsible for the malignant transformation of HPV-infected cells. As previously noted, the E6 gene is poorly conserved and highly variable. The evolutionary variability of E6 is demonstrated by the fact that not all papillomaviruses even code for E6; the γ-HPVs HPV101 and HPV103, for example, code for the E10 protein instead [88,89]. The differences between α- and β-E6 oncoproteins are, therefore, expected. However, some homology exists, and this is reflected among a subset of the commonly shared targets between the oncoproteins (Figure 2). The more interesting fact is that there are several other E6 targets, which are genus-exclusive, and thus affected in distinct and specific ways by each of the oncoproteins (Figure 2). The interplay between E6 oncoproteins and these particular substrates is likely to be responsible for the differences among different HPVs in their viral fitness and oncogenic potential.

### 5.1. Apoptosis

#### 5.1.1. p53 

p53 is probably the most well-known and extensively researched tumor suppressor protein. It serves as “the guardian of the genome”; that is, its main function is the maintenance of the genome integrity of the cell, but it also plays a part in development, differentiation, and aging [90]. As a response to DNA stress (replication stress, DNA damage, or oncogene activation), p53 undergoes a post-translational modification, prolonging its half-life and enabling it to bind to specific DNA sequences in the cell. This promotes the transcription of genes involved in various biological processes, ultimately leading to cell cycle arrest, senescence, DNA repair, or apoptosis [90]. Due to the importance of its roles, it is clear why p53 is mutated or inactivated in over half of all human cancers, including HPV-induced malignancies.

**Figure 3:**
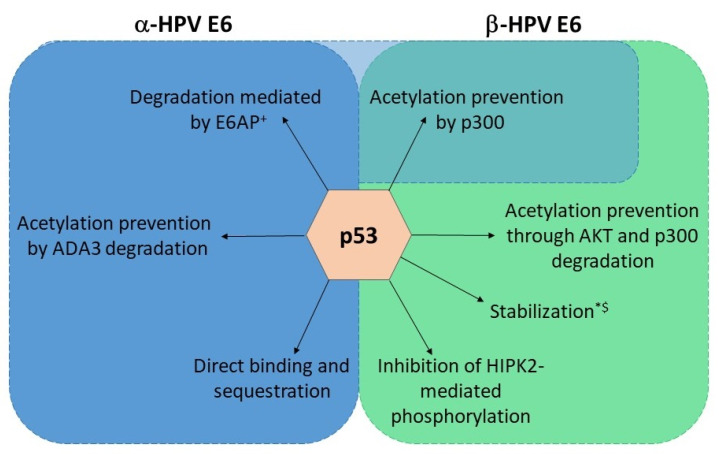
Strategies employed by E6 oncoproteins in manipulating p53 signaling. α- and β-E6 oncoproteins mostly have distinct ways of affecting p53. The ones typical for α-E6 are indicated in the blue box, and the ones typical for β-E6 in the green one. The mechanism shared between both E6 oncoproteins is indicated by the overlapping area. * denotes that the claim refers to β2 species, ^+^ that it refers to β3 and ^$^ that it refers to β4.

HR HPV E6 oncoproteins interact with the ubiquitin ligase E6-associated protein (E6AP) of the host via the LXXLL motif, resulting in E6/E6AP complex, which ensures E6 protein stability in the cell [91,92]. This interaction then further enables the oncoprotein to form a ternary complex with p53, leading to p53 proteasomal degradation and the evasion of cell death (Figure 3) [93]. LR HPV E6 oncoproteins do not show this ability, even though they can bind E6AP [94,95]. Additionally, α-HPV E6 oncoproteins have developed several additional mechanisms for inhibiting p53 signaling. E6 can prevent p53 acetylation via the binding of the CBP (CREB-Binding Protein)/p300 histone acetyltransferase and the formation of a p53-E6-p300/CBP complex (Figure 3) [96,97,98]. p53 acetylation is also inhibited via the E6-mediated degradation of the transcriptional coactivator ADA3 (Alteration/Deficiency in Activation 3) (Figure 3) [99]. Signaling via p53 can be inhibited by the direct binding of E6 to p53, and both LR and HR HPVs show this ability [100,101]. Finally, E6 has the ability to sequester p53 in the cytoplasm, thus disabling its effects (Figure 3) [102]. 

As already mentioned, the ability of HR-HPV E6 to degrade p53 depends on its interaction with E6AP. On the contrary, the vast majority of β-HPV E6 does not interact with E6AP and degrade p53 even though they have the same LXXLL binding motif. Very notable exceptions to this “rule” are β3 species types HPV49, HPV75, and HPV 76, which bind E6AP and degrade p53, the β2 species HPV38 and β4 species HPV 92, which bind E6AP but stabilize p53, and a β1 species HPV24 that binds E6AP, but its effect on p53 turnover has not been evaluated (Figure 3) [70,103,104,105]. However, it is broadly considered that β-HPV E6 proteins convey resistance to apoptosis and interfere with p53 functions by inhibiting its post-translational modifications [79]. Similar to α-E6 oncoproteins, β-HPV E6 oncoproteins can interact with p300/CBP and prevent the acetylation of p53 (Figure 3) [80]. They also have an additional mechanism for reducing p53 acetylation by p300/CBP—the inhibition of p300 stabilization by AKT and its subsequent degradation in a proteasome-dependent manner (Figure 3) [106]. This leads to a delayed accumulation of p53, increased resistance to apoptosis, and the persistence of DNA damage induced by UVB radiation [107]. HPV38, again, seems to be an exception; the E6 oncoprotein of this HPV type weakly binds p300 but does not induce its degradation. Nevertheless, this interaction seems to be important for the inhibition of p53-dependent apoptosis [80]. Finally, β-HPV E6 prevents the HIPK2 (Homeodomain-Interacting Protein Kinase 2)-mediated phosphorylation of p53 in keratinocytes by binding the regulator and, possibly, inhibiting its interaction with p53 (Figure 3) [108]. 

The role of p53 in the cell and its importance for maintaining cellular integrity has long been known. In the context of viral infections, this is additionally demonstrated by the fact that p53 is one of the rare common targets of HPVs, regardless of the genera. This suggests that abrogating p53 function is key for the virus to survive and thrive within the host cell, which is commonly carried out by several different, genus-specific mechanisms.

#### 5.1.2. BAK and BAX 

BAK (Bcl-2 homologous Antagonist/Killer) and BAX (BCL2 Associated X, Apoptosis Regulator) are members of the proapoptotic branch of the Bcl-2 family of proteins. As a response to apoptotic stimuli, BAK and BAX are activated and accumulate in the outer mitochondrial membrane. There, they oligomerize and mediate membrane permeabilization, which leads to the release of other proapoptotic factors and the progression of apoptotic signaling [109]. This event is considered a point of no return in programmed cell death, and BAK and BAX are thought of as indispensable for the process. 

α-HPV E6 oncoproteins degrade BAK in a manner similar to p53, i.e., by utilizing the E6AP ubiquitin ligase [110]. This ability is not limited to only HR types but is also found in LR ones, albeit to a lesser extent, corresponding to their reduced ability to prevent apoptosis [111]. BAX was also found to be degraded and downregulated in keratinocytes expressing E6 [112]. It was initially thought that BAX downregulation happened as a consequence of p53 degradation by E6, but this does not necessarily seem to be the case, as it was also found in cells lacking p53. Regardless, the precise mechanism of the inhibition of *BAX* expression is still not clear [112,113]. In addition to BAK and BAX, E6 can stimulate the degradation of the FADD (FAS-Associated Death Domain) receptor and downregulate procaspase 8, both of which are important for triggering and conducting apoptotic pathways (Figure 2) [75]. 

BAK seems to be especially important when it comes to UVB-induced DNA damage in the skin, which is why the ability of β-E6 to degrade BAK has extensive consequences [114]. All β-HPV types seem to have this ability and, like with α-E6 oncoproteins, the degradation is proteasome and ubiquitin ligase dependent, although there are some discrepancies in regard to which ubiquitin ligase is needed [115,116]. β-HPV E6 oncoproteins also seem to prevent the accumulation of BAK and BAX in infected skin keratinocytes after UV irradiation [117,118]. Interestingly, not all β-E6 oncoproteins seem to be equally capable of preventing UV-induced apoptosis, but the specific reason for this is currently unknown [117]. To corroborate the importance of these interactions, several reports note that decreased levels of BAK have been found in HPV-associated skin cancers when compared to HPV-negative ones [11,118,119]. It is worth noting that there is some indication that HPV8 E6 also interacts with FADD, but not much is known about this [120]. 

Apoptosis resistance is clearly important for the successful viral replication in infected cells, but what is particularly interesting in the case of HPV E6 oncoproteins is the fact that both α and β-E6s target the same components of this vast apoptotic cascade. While α-E6s have additional targets involved in programmed cell death, BAK and BAX degradation are clearly central and universal in the process of viral replication and consequent viral pathogenesis.

#### 5.1.3. hTERT

Telomeres are repetitions of the 5′-TTAGGG-3′ sequence, 5000 to 15,000 nucleotides in length, found on the ends of chromosomes. They are shortened by each cell division and reaching the critical telomere length triggers programmed cell death. For this reason, actively dividing cells employ the enzyme telomerase to extend their telomeres. Telomerase is composed of the TERC (Telomerase RNA Component; a sequence template), hTERT (Telomerase Reverse Transcriptase), and several auxiliary proteins (the dyskerin complex). The TERT gene is upregulated or activated in 85–95% of all human cancers, and the same can be postulated about HPV-induced malignancies [121].

HR-HPV E6 has multiple mechanisms for telomerase activation. Firstly, E6 directly interacts with hTERT and telomeric DNA, and this activity appears to be E6AP-dependent [86,122]. Secondly, it regulates the hTERT promoter and participates in epigenetic and post-transcriptional hTERT regulation [123]. The E6/E6AP complex interacts with c-Myc, an hTERT expression regulator, and helps it displace the hTERT repressor complex, resulting in increased hTERT gene transcription [122,124,125]. Additionally, an hTERT repressor, NFX1-91 (Nuclear Transcription Factor, X-box Binding 1), undergoes degradation in an E6AP-dependent manner, leading to an increase in HAT (Histone Acetyltransferase) activity and a decrease in HDAC (Histone Deacetylase) activity, and ultimately to additional hTERT transcription [126,127]. Interestingly, another splicing variant of the same protein, NFX1-123, interacts with HR-HPV E6, and together, they bind and stabilize hTERT mRNA, further amplifying the effect of hTERT upregulation [127,128,129,130]. The positive impact of NFX1-123 has also been corroborated in primary CC samples and HPV+ HNSCC [129,131,132]. Furthermore, in vitro studies have revealed alterations in DNA methylation patterns during HPV infection in HPV+ cells. These changes include hypermethylation of specific hTERT promoter regions and hypomethylation of other regions in cells with HR-HPV oncoproteins [133]. This is at least partially due to the interaction of HR-E6 with KDM5C (Lysine Demethylase 5C) demethylase of H3K4 histone and its subsequent degradation [134]. 

Thus far, only HR α-HPVs have been definitely proven to activate hTERT, but, surprisingly, some β-HPV E6 oncoproteins seem to also show this ability [135,136]. This appears to be dependent on the strength of the interaction between the oncoproteins and E6AP or NFX1-91 and is, therefore, found only in β-types that show the capacity for cellular transformation, such as HPV38 and HPV49 [70,135,137]. 

Because telomerase shortening is a safeguard from uncontrolled cell division, and HPVs induce physiologically unnecessary proliferation of keratinocytes, the virus had to develop means to circumvent this mechanism. This appears to be the characteristic of the virus types that exhibit a greater potential for cell transformation, and this is probably at least in part due to hTERT activation. As a consequence, hTERT activation is also a hallmark of HPV-induced cancers, as it is of many other human cancers. 

### 5.2. DNA Damage Repair 

To repair single- or double-strand breaks in the DNA, the cell utilizes several, often overlapping, mechanisms. The mechanism used to repair single-strand breaks is called single-strand break repair, while double-strand breaks are repaired by non-homologous end joining and homologous recombination [138]. The most common proteins participating in these processes belong to the phosphatidylinositol 3-kinase-like protein kinase (PIKK) family. These are ATM, ATR (ATM- and Rad3-Related), DNA-PKca (DNA-dependent Protein Kinase), and various members of the PARP (Poly (ADP-Ribose) Polymerase 1) family of proteins. ATM and DNA-PKcs detect double-strand breaks caused by DNA-damaging agents, while ATR detects long single strands of DNA [138]. After being recruited to break sites on the DNA, the proteins are activated by phosphorylation, after which they phosphorylate downstream targets [138]. 

Constitutively active DNA damage repair pathways have been found in cells expressing cancer-causing HPVs, even in the absence of DNA-damaging agents, and there is evidence indicating that this is crucial for viral replication [76,139]. The activation of DNA repair pathways, especially following treatment with chemotherapeutic drugs, has an impact on HR-E6 binding preferences—the activation leads to E6 PBM phosphorylation and a switch in binding partners (to be discussed later) [140,141]. HPV oncoproteins have been found to co-localize with various members of the repair pathways on host and viral genomes, and abrogating the pathways by either silencing or with inhibitors leads to a decrease in differentiation-dependent viral genome amplification [142,143]. The hijacking of DNA repair proteins by HPV and the preferential binding of these proteins to viral DNA leads to enhanced levels and accumulation of DNA breaks, possibly resulting in HPV genome integration and host genome instability [144]. The manipulation of DNA repair mechanisms is usually attributed to the actions of HR-E7, but E6 also contributes to the process. Epithelial cells expressing HR-E6 were found to have a reduced capacity for repairing UV-induced thymidine dimers [145,146]. E6 also reduced the rate and fidelity of DNA end joining in both p53-dependent and independent ways [147]. One of the lesser-known targets of HR-E6 is the protein MGMT (O-6-Methylguanine-DNA Methyltransferase) (Figure 2). This protein is a methyltransferase that removes the alkyl group covalently bonded to DNA, thereby preventing mutations, aberrations, and breaks [148]. E6 degrades MGMT in an E6AP-dependent manner and, by doing this, disables another DNA repair pathway [149]. Finally, E6 binds XRCC1 (X-ray Repair Cross Complementing 1), a part of a multiprotein complex necessary for repairing single-strand breaks, which leads to a decrease in DNA repair efficiency [150,151]. α-E6s share this ability with their β counterparts, albeit to a much greater extent [150].

The repairs of single and double-strand breaks are also significantly delayed in keratinocytes infected with β-HPV, but β-oncoproteins manipulate these pathways in a distinct way [82,107]. Both single and double-strand damage repair are thought to be disrupted by the β-E6 degradation of p300 [152,153]. As mentioned, p300 degradation leads to the reduction in ATR levels, indispensable for single-strand break repair, but also to the downregulation of BRCA1 and BRCA2 (BRCA1 and BRCA2 DNA Repair Associated), important for the homology repair of double-strand breaks [82,106,107]. It is tempting to speculate that α-E6 could also modulate damage repair systems in the same manner by their well-known degradation of p300, but, as to our knowledge, no proof of this seems to exist. Furthermore, β1 HPV8 E6 attenuates the activity of DNA-PK, a protein kinase indispensable for non-homologous break repair, and decreases the available amount of ATM and ATR in infected keratinocytes [152,154]. These results are corroborated by in vivo studies that demonstrate the physiological relevance of this inhibition [155].

### 5.3. Differentiation

#### 5.3.1. Notch Signaling 

The Notch signaling pathway is a conserved pathway involved in the development of various tissues. It is dependent on direct contact, initiated by the interaction of Notch receptors (NOTCH1-4) on one cell and ligands (JAGGED1, JAGGED2, DLL1, DLL3, and DLL4) on another. The interaction induces proteolytic cleavages of the receptor, which results in the release of the Notch intracellular domain (NICD). The NICD translocated to the nucleus, associates with the DNA-binding protein RBPJκ, MAML1 (Mastermind-like Transcriptional Coactivator 1), and other co-activators to form a Notch transcriptional factor complex. The complex then binds to Notch-responsive elements and activates the transcription of downstream target genes [156].

Notch signaling exerts multifaceted control over the developmental progression of basal keratinocytes, safeguarding their undifferentiated state, preserving the compactness of the proliferative skin compartment, and directing their commitment to the epidermal lineage. It is unsurprising that HPV oncoproteins would interfere in this process. Activated Notch signaling exerts its influence on differentiating keratinocytes by inducing the expression of early differentiation markers [157]. This intricate signaling cascade also stimulates the expression of p21WAF1/CIP1 and caspase 3, leading to growth suppression and the cessation of proliferative signals in differentiating cells [158]. Consequently, the activation of canonical Notch signaling in the suprabasal cell layers of the epidermis becomes tantamount to a commitment to the epidermal lineage [159]. In the process of differentiation, p63 works antithetically to Notch1. Elevated p63 expression counteracts Notch1-induced growth suppression, while active Notch signaling leads to p63 downregulation, underscoring the significance of this equilibrium in epidermal maintenance and control [160].

This pathway is one of the rare instances where the impact of α-HPV E6 oncoproteins is less clear than those of β-HPV, not for the lack of trying. HR E6 oncoproteins induce elevated Notch signaling via the upregulation and post-transcriptional editing of Notch components, as well as NOTCH1 protein localization [161,162]. The upregulation of NOTCH1 expression is thought to occur via a p53- or p63-dependent mechanism or the NFX1-123 splicing variant, as mentioned above (Figure 2) [163,164,165,166]. Interestingly, a recent study has shown that NFX1-123 could even impact keratinocyte differentiation independently of Notch signaling (Levan 2020). The increased Notch activation translates to an increase in proliferation as well as tumorigenicity of CC cells [161]. Nevertheless, there is conflicting evidence pointing toward a completely different conclusion. Increased Notch1 activity was found to downregulate E6 expression, and overexpression of the NOTCH1 receptor in HPV18+ CC cell line HeLa leads to cell growth arrest [167,168,169]. To make things even more confusing, both activated and suppressed Notch signaling have been found to be a factor in CC progression, even though the evidence is stronger in favor of the oncogenic function of Notch [162,170,171,172]. 

Unlike α-HPVs, the situation of Notch in β-HPV infections is straightforward. The E6 oncoprotein of β-HPV types binds the MAML1 cofactor and inhibits its association with NICD, thereby abrogating Notch signaling (Figure 2). As a consequence, p21 is not transcribed, and the cell cycle continues [173,174,175]. β-E6 binds MAML1 via the LXXLL motif—the same motif α-E6 utilizes to interact with E6AP [176]. This binding partner preference was even found to cluster different E6 proteins into genera, but it is not completely exclusive [177]. The E6 oncoproteins of species in the β-3 group (HPV49, HPV75, and HPV76) were previously found to bind both MAML1 and E6AP (Figure 2) [70], while our group recently demonstrated that HR E6 oncoproteins from types 16, 18 and 33 can also bind MAML1, which results in their protein stability (Figure 2). The consequence of this interaction appears to result in increased proliferation and migration of HPV+ CC-derived cell lines [178]. An additional way to abrogate keratinocyte differentiation is via the induction of ΔNp63α, a Notch antagonist. ΔNp63α is upregulated by the inhibition of miR-203, its negative regulator. miR-203, on the other hand, is inhibited by the downregulation of c/EBPα, which happens as a consequence of p300 degradation by β-E6 [179,180]. The ultimate effect of all of this is the delayed differentiation and prolonged proliferation of basal keratinocytes. 

The Notch signaling pathway plays a pivotal role in the regulation of basal keratinocyte development and epidermal maintenance, and its mechanisms are deeply intertwined with the life cycle of HPVs. In the skin, the effect of E6 on Notch signaling and, subsequently, on the differentiation of epithelial cells is obvious and unequivocal, while the precise effects in the mucosa are yet to be delineated. Regardless, it is apparent that Notch is important for the life cycles of both α and β-HPVs.

#### 5.3.2. TGF-β

Transforming growth factor-β (TGF-β) is an evolutionarily conserved pathway controlling tissue homeostasis. A variety of ligands can trigger its activation; the receptor is a heterotetrameric molecule with serine/threonine kinase activity, TGF-β, consisting of one TGFBR2 dimer and one TGFBR1 dimer. Receptor-associated SMAD (R-SMAD) proteins are bound to the receptor, and downstream of them are located co-activator SMAD proteins (co-SMAD). Different R-SMADs are activated and phosphorylated depending on the ligand and receptor in question. The phosphorylated R-SMADs complex together with the co-SMAD, enter the nucleus and act as transcription factors [181]. They can activate the expression of proteins involved in cell cycle arrest, like p21 and p27, but also of proteins active during epithelial-mesenchymal transition and cell invasion [181,182,183]. β-E6 oncoproteins have the ability to bind SMAD2, SMAD3 (R-SMADs), and SMAD4 (co-SMAD) but do not destabilize them or induce their degradation. However, they do decrease the formation of the SMAD2/3/4 complex and its binding to target DNA (Figure 2) [184]. In this manner, β-E6s inhibit TGF-β signaling. 

Interestingly, α-E6 oncoproteins do not seem to have this ability, but α-HPVs can inhibit TGF-β signaling via the functions of E5 and E7 [185,186]. The mechanism of α-E7 signaling is, in fact, remarkably similar to that of β-E6 [187]. There is a limited amount of evidence pointing toward the degradation of the PDZ domain-containing protein TIP-2/GIPC by HPV18 E6, which leads to a modulation in the expression of TGF-β receptors and an overall decrease in the cytostatic effect of TGF-β signaling (Figure 2) [188].

Taking all of this together, TGF-β seems to be another well-conserved target of mucosal and cutaneous HPVs. The fact that the mechanism of TGF-β inhibition is regulated by different viral oncoproteins, depending on the genus, confirms the importance of this function for the viral life cycle. 

### 5.4. The Immune Response

In order to establish a successful infection and sustain viral production, HPVs employ various evasion strategies to avoid immune detection. Firstly, the exposure of HPV antigens to the immune system is limited because they are not present throughout the host system. Secondly, genes regulated by the early promoter are expressed within basal epithelial cells and in low copy numbers [189]. This restricts their visibility to the immune system. Furthermore, unlike many other viruses, HPV does not induce cell lysis, reducing the probability of antigen-presenting cells (APCs) encountering virions and subsequently presenting them to immune cells [189]. A noteworthy evasion tactic employed by HPV involves the expression of its highly immunogenic late proteins predominantly in the cornified epithelium, where immune cell presence is sparse [190]. Nevertheless, it is important to recognize that keratinocytes, which constitute the cornified epithelium, serve an essential role in immune surveillance and are regarded as a type of non-specialized antigen-presenting cells (APCs). These keratinocytes express pathogen recognition receptors (PRRs) that can detect the presence of microbial agents, thereby initiating innate and adaptive immune signaling cascades [191]. 

HPV oncoproteins have evolved to modulate several components important for immune signaling. In α-HPV infections, the immune response is modulated mostly due to the effects of E5 and E7, but E6 also plays a role. In antiviral immune responses, interferon (IFN) plays an important part [192]. IFN expression can be initiated by the activation of PRRs and their triggering of IFN regulatory factors (IRFs) [193]. HPV16 E6 binds IRF3 and prevents its translocation to the nucleus, effectively blocking the expression of IFN-I [194]. The JAK-STAT (Janus Kinase/Signal Transduction and Transcription Activation) signaling pathway is the central communication node of the immune system, and it can be triggered by any member of the IFN family [195]. HR-HPV oncoproteins interfere with JAK-STAT signaling in a number of ways, among which is the direct downregulation of STAT1 by transcription inhibition [196]. This inhibition is thought to be important in early viral replication [189]. Conversely, activated JAK-STAT signaling can be found in HPV-induced malignancies. A probable reason for this is the positive influence of STAT3 and STAT5 on keratinocyte proliferation and the inhibition of their differentiation [197,198,199]. Indeed, STAT3 expression was found to be crucial for the completion of the viral life cycle of HPV18, and 18 E6 was shown to have the ability to induce STAT3 activation (Figure 2) [139,200]. The prolonged effects of HPV oncoproteins during persistent infection and the changes in gene expression they cause do not only impact the infected cells themselves but the entire microenvironment. Fewer APCs are recruited and retained, the amount of pro-inflammatory cytokines increases, and there is a change in the number and type of immune cells infiltrating the tumor. Taken together, all these changes create a pro-inflammatory microenvironment that allows for tumor progression while simultaneously disabling the proper immune response. 

As described above, β-HPVs were first discovered in skin lesions of immunocompromised people, indicating that the immune system of a healthy individual keeps the β-HPVs from exhibiting pathological effects and causing cellular transformation [201,202]. When this safeguard is removed, β-HPVs can act as cofactors in carcinogenesis. They modulate cellular pathways to ensure their own propagation, and, as a consequence, they make the cell more susceptible to UV damage (hit-and-run hypothesis) (Figure 1B) [203]. UB-B damage has even been found to stimulate the promoter activity of HPVs [204]. In the case of HPV8, IRF7 was involved in this upregulation [205]. In opposition to this, IRF3 was found to suppress HPV8, but unlike HPV16, HPV8 E6 only weakly antagonized IRF3 by binding to its partner CBP (Figure 2) [205,206]. The inflammatory response of the skin was found to vary between HPV types—HPV5 oncoproteins were found to act more pro-inflammatory than those of HPV38, a known transforming agent [207]. However, HPV38 caused a much higher pro-inflammatory response in UVB-irradiated keratinocytes when compared to non-infected cells [208]. NFκB (Nuclear Factor Kappa B) was also found to be more activated in HPV38-infected UVB irradiated keratinocytes, contributing to their survival [209]. UV irradiation can also stimulate the expression of Toll-like receptor 9, a PRR involved in the defense against bacteria and viruses. HPV38 oncoproteins can block the expression and activation of TLR9 [210,211]. 

Most of what is known about the interaction of the immune system and β-HPVs is deduced from findings in EV patients. In fact, β-HPVs are the only pathogen typical EV patients are more susceptible to [212]. These people carry the mutations for EVER1 and EVER2—transmembrane, channel-like proteins 6 and 8 expressed in the ER and are suspected to be involved in the regulation of Zn levels in keratinocytes and possibly immune cells [203,213]. The loss of EVER2 was found to impair NF-κB signaling and activate the transcription of HPV5 via a JNK (*c-*Jun N-terminal Kinase**)-dependent pathway [214]. Another candidate for the protein mediating keratinocyte immune resistance to β-HPVs is CIB1 (Calcium and Integrin Binding 1) because this genus does not code for a protein to neutralize it [212]. In addition, CIB1 is downregulated in EV patients. Moreover, severe combined immune deficiency patients carrying a JAK3 mutation (JAK-STAT signaling), or a mutation of the common gamma chain (subunit of 6 different interleukin receptors) are also susceptible to β-HPV infections and EV-like disease (atypical EV). All of the aforementioned indicates that β-HPV immunity must be conveyed via these proteins, but more research is needed before any conclusions can be drawn. It also seems that β-HPV infection causes a chronic inflammatory response, which may promote skin carcinogenesis. 

## 6. α- and β-HPV E6 Specific Targets

The aforementioned processes and cellular targets are, of course, some of the ones that are most important for the viral life cycle and the subsequent process of carcinogenesis, but there are also a number of protein interactions unique for the HPV types of a particular genus (Figure 2). Some of these may hide a key for revealing the differences in carcinogenic potential of the different virus types. 

There are several targets unique to the E6 oncoproteins of HR-HPVs, the most significant of which are probably PDZ domain-containing proteins (Figure 2). PDZ domains are protein–protein interaction motifs, 80–110 aa in length, and found on about 150 distinct human proteins, most of which contain multiple copies of the motif [215]. The PDZ (PSD95/Dlg/ZO-1) class I binding motif (PBM) is usually found on the C-terminus of HR-HPV E6 proteins, and it can be highly variable, with 16 subtypes proposed [87,216]. All HR-HPV E6 proteins contain the X-S/T-X-Φ_COOH_ consensus site important for PDZ binding, but they also display significant variation within the region. In some cases, the subtle variations within the PBM can determine substrate preferences [36,217]. There are currently 19 PDZ domain-containing proteins for which the E6 interaction has been confirmed, and they participate in a variety of cellular processes [87]. Some of the most prominent E6 binding partners are proteins involved in the regulation of cell–cell contacts and cell polarity maintenance, such as SCRIB (Scribble Planar Cell Polarity Protein), DLG1 (Discs Large MAGUK Scaffold Protein 1), and MAGI1/2/3 (Membrane Associated Guanylate Kinase, WW and PDZ Domain Containing 1/2/3) [218,219]. E6 targets these and other PDZ domain-containing proteins for proteasome-mediated degradation, thereby disrupting cell polarity. Additionally, E6 can disrupt cellular homeostasis and proliferation by targeting, for example, the proteins SNX27 (sorting nexin 27) and CAL (Golgi Associated PDZ and Coiled-coil Motif Containing), important for the trafficking of proteins, or proteins NHERF1 and NHERF2, which act as protein scaffolds and negative regulators of epithelial proliferation [87,95,220,221,222]. The interactions E6 achieves via the PBM are indispensable for both the viral life cycle and for the tumorigenesis of the virus; mutating the PBM significantly reduces viral replicative potential, as well as abolishes the viruses’ potential for keratinocyte transformation [223,224,225]. 

In a surprising twist, a PDZ domain-containing protein, syntenin-2, was found to be downregulated in HPV8 E6 expressing cells [226]. However, this finding was not attributable to protein–protein interactions via the PBM but to syntenin-2 protein hypermethylation [83]. Likewise, there is evidence that β2 HPV38-E6 binds the PDZ domain-containing protein PTPN13 (Protein Tyrosine Phosphatase Non-receptor type 13), but there is little known about this interaction and its significance [104,227].

Interestingly, HR-HPV E6 proteins utilize the same PBM motif for interacting with the 14-3-3 family of proteins (Figure 2) [228,229]. The members of this protein family are adapter proteins, which interact with a variety of cellular proteins involved in several processes [230]. The switch between the PDZ domain-containing proteins and the 14-3-3- family happens in a phosphorylation-dependent manner due to the actions of PKA (Protein Kinase A) or AKT [231]. This directly links the DNA damage repair system and the ability of E6 oncoproteins to modify p53 activity [141]. DNA damage pathways cause the CHK1- and CHK2-(Checkpoint Kinase 1 and 2) dependent phosphorylation of the E6 PBM; E6 then interacts with 14-3-3, and p53 transcriptional activity is abrogated [141]. This is particularly intriguing when considering that p53 activity and cellular distribution are linked to and dependent on particular 14-3-3 isoforms [232]. Because the members of 14-3-3 are versatile, the possibilities of the interaction between E6 and 14-3-3 impacting cell behavior are vast and could also be dependent on the phosphorylation and isoform of 14-3-3. For now, the best described and seemingly preferred interaction is that between 14-3-3 ζ and E6, which increases E6 stability in HeLa cells [228]. 

As previously mentioned, E6 oncoproteins interact with and execute their functions via several ubiquitin ligases, with HECT domain-containing E3 ubiquitin ligase E6AP being the most prominent one for α-E6s [233]. Furthermore, HR 18 E6 was shown to interact with another HECT domain-containing E3 ubiquitin ligase EDD, independently of E6AP. The alterations in EDD levels were shown to affect E6/E6AP complex proteolytic activities and, in this way, impact the degradation of E6 cellular substrates, such as p53 [233]. HR-E6 also interacts with BRCA1 and BARD1 (BRCA1 Associated RING Domain 1) ubiquitin ligases, which also serve as tumor suppressors (Figure 2) [234,235]. The details of these interactions are not clear, but there is speculation that HR-E6 antagonizes BRCA1 [235]. Similarly, β2-E6 oncoproteins uniquely interact with ubiquitin ligases, as well. Notably, β2-E6 interacts with members of a multiprotein complex CCR4 (C-C Motif Chemokine Receptor 4)-Not an evolutionarily conserved complex involved in regulating mRNAs (Figure 2) [104,219]. Its precise functions and the impact of its interaction with β2-E6 are currently unknown [236]. As hinted previously, β-E6 oncoproteins use a ubiquitin ligase to degrade BAK, but the specific ligase being used is still debated. First, reports claimed that E6AP was needed for the degradation. Still, subsequent analysis showed that, at least in the case of HPV5, a unique binding partner, the ligase HERC1 (HECT and RLD Domain-containing E3 Ubiquitin Protein Ligase Family Member 1) specifically targets BAK in the presence of HPV5-E6 (Figure 2) [115,116]. Another very interesting ubiquitin ligase, p600, was also found to bind to the β2 HPV38 E6 [104,105]. This is particularly fascinating when considering that p600 is a typical binding partner of HR and LR HPV E7 proteins [237]. One particular β-HPV was found to have a number of unique binding partners—HPV92 is the only member of the β4 species, and, likewise, its E6 protein is particular in its interactions [104,238]. Notably, HPV92-E6 binds to both subunits of a HIF1 (Hypoxia Inducible Factor 1) heterodimer, a transcription factor composed of a constitutive β subunit and a hypoxia-inducible α subunit. Additionally, HPV92 E6 binds components of the centrosome (CEP131, CEP152, CEP63, and CEP44), several proteins involved in cell adhesion (JUP, AAMP and Paxilin), the microtubule-binding protein KIAA1543, a negative regulator of MAPK signaling (DUSP3) and a cation transporter involved in keratinocyte proliferation (SLC12A8) (Figure 2) [104]. All of this corroborates the distinct phylogenic development of the β4 species.

This is not an exhaustive list of all unique interacting partners of α- and β-E6 oncoproteins, but it describes some of the more significant or interesting ones. A great deal more information is available for α-E6 oncoproteins, and, in this regard, the authors took the liberty to highlight the interactions that are described in the literature to a more detailed extent. β-E6 oncoproteins, on the other hand, are much less researched, and the individual species of the genera are much more distinct. β-HPV diversity is, again, evident from the differences in E6 binding partners between species that can sometimes be more pronounced than the differences between genera. For these reasons, and in the interest of fairness, the interacting partners of different β-E6 oncoproteins were covered, even when they were specific to the type or species.

## 7. Conclusions

Studies investigating α- and β-HPV E6 oncoproteins have provided valuable insights into the complex mechanisms underlying viral oncogenesis and their role in the development of cervical and cutaneous cancers, respectively. While E6 oncoproteins from both mucosal and cutaneous types share some common features, such as their ability to target p53, BAK, and interact with some components of the ubiquitin–proteasome system, they also exhibit significant differences in their modes of action and biological functions. Nevertheless, both α- and β-HPV E6 proteins underscore the significance of viral oncoproteins in modulating the key processes within the infected cells—apoptosis, DNA damage repair, proliferation, and differentiation—and in their surroundings. All of the aforementioned could be considered of vital importance for viral propagation and, as a consequence, for the onset of carcinogenesis. Some unique interacting partners, on the other hand, could show us what particular modifications are needed to achieve optimal viral replication at different anatomical sites or what interactions bear a greater potential for cell transformation. Comparing the E6 oncoproteins of the species in both genera helps in clarifying the hierarchy of the importance of their interactions and the role they pose in cell transformation. Establishing this could lead to a better prioritization in developing potential therapeutics and forming a more complete and direct approach to therapy, as well as broadening the possibilities for the treatment of HPV+ cSCCs.
